# Plan comparison of volumetric-modulated arc therapy (RapidArc) and conventional intensity-modulated radiation therapy (IMRT) in anal canal cancer

**DOI:** 10.1186/1748-717X-5-92

**Published:** 2010-10-13

**Authors:** Sabine Vieillot, David Azria, Claire Lemanski, Carmen Llacer Moscardo, Sophie Gourgou, Jean-Bernard Dubois, Norbert Aillères, Pascal Fenoglietto

**Affiliations:** 1 Département de Cancérologie Radiothérapie et de Radiophysique, CRLC Val d'Aurelle-Paul Lamarque, Montpellier, France; 2 Unité de Biostatistiques, CRLC Val d'Aurelle-Paul Lamarque, Montpellier, France

## Abstract

**Background:**

To compare volumetric-modulated arc therapy (RapidArc) plans with conventional intensity-modulated radiation therapy (IMRT) plans in anal canal cancers.

**Methods:**

Ten patients with anal canal carcinoma previously treated with IMRT in our institution were selected for this study. For each patient, three plans were generated with the planning CT scan: one using a fixed beam IMRT, and two plans using the RapidArc technique: a single (RA1) and a double (RA2) modulated arc therapy. The treatment plan was designed to deliver in one process with simultaneous integrated boost (SIB) a dose of 59.4 Gy to the planning target volume (PTV2) based on the gross disease in a 1.8 Gy-daily fraction, 5 days a week. At the same time, the subclinical disease (PTV1) was planned to receive 49.5 Gy in a 1.5 Gy-daily fraction. Plans were normalized to 99% of the PTV2 that received 95% of the prescribed dose. Planning objectives were 95% of the PTV1 will receive 95% of the prescribed dose and no more than 2% of the PTV will receive more than 107%. Dose-volume histograms (DVH) for the target volume and the organs at risk (bowel tract, bladder, iliac crests, femoral heads, genitalia/perineum, and healthy tissue) were compared for these different techniques. Monitor units (MU) and delivery treatment time were also reported.

**Results:**

All plans achieved fulfilled objectives. Both IMRT and RA2 resulted in superior coverage of PTV than RA1 that was slightly inferior for conformity and homogeneity (p < 0.05).

Conformity index (CI_95%_) for the PTV2 was 1.15 ± 0.15 (RA2), 1.28 ± 0.22 (IMRT), and 1.79 ± 0.5 (RA1). Homogeneity (D_5% _- D_95%_) for PTV2 was 3.21 ± 1.16 Gy (RA2), 2.98 ± 0.7 Gy (IMRT), and 4.3 ± 1.3 Gy (RA1). RapidArc showed to be superior to IMRT in terms of organ at risk sparing. For bowel tract, the mean dose was reduced of 4 Gy by RA2 compared to IMRT. Similar trends were observed for bladder, femoral heads, and genitalia. The DVH of iliac crests and healthy tissue resulted in comparable sparing for the low doses (V10 and V20). Compared to IMRT, mean MUs for each fraction was significantly reduced with RapidArc (p = 0.0002) and the treatment time was reduced by a 6-fold extent.

**Conclusion:**

For patients suffering from anal canal cancer, RapidArc with 2 arcs was able to deliver equivalent treatment plan to IMRT in terms of PTV coverage. It provided a better organ at risk sparing and significant reductions of MU and treatment time per fraction.

## Background

Conventional chemoradiation is the established treatment for anal carcinoma. This organ-preserving approach gives an equivalent cure than radical surgery but at the cost of high acute and late pelvic toxicities. These side-effects can lead to undue treatment breaks and long overall treatment times and therefore may negatively influence outcome [1-3].

Intensity-Modulated Radiotherapy (IMRT) is a treatment delivery technique based on inverse planning optimisation to modulate intensity beams by using multileaf collimator (MLC). During radiation delivery, the leaves are adjusted while the beam is on. IMRT allows the possibility of producing concave dose distributions and providing specific sparing of normal tissue [4]. We performed a dosimetric study about anal canal carcinoma and showed that IMRT resulted in significant reductions in the doses delivered to the bowel, bladder and genitalia/perineal skin [5]. These dosimetric findings were correlated with lower rates of acute GI and GU morbidities and high conformation to the target volume for anal carcinoma [6-10].

We started to treat patients suffering for anal carcinoma with IMRT in May 2007 but we rapidly switch to volumetric modulated arc therapy (VMAT). Indeed VMAT is a new form of IMRT optimisation combining one gantry rotation and the following capabilities: variable dose-rate, variable gantry speed and dynamic MLC [11]. Details of the RapidArc process and quality assurance are detailed in several publications [11,12]. The VMAT approach has a number of potential advantages compared to IMRT: reducing significantly the treatment time and the number of MU, improving normal tissue sparing while keeping the adequate coverage.

In the present study we compared RapidArc with IMRT in anal canal patients including iliac crests sparing measurements.

## Methods

### Patient selection, simulation and treatment planning

Ten patients with localized anal canal carcinoma treated with IMRT in our institution were selected for this study. Five patients were staged II, three IIIA, and two IIIB according to the American Joint Committee on Cancer 2006 Guidelines (AJCC) [13]. Details are shown in Table 1.

**Table 1:** Tumor staging, PTV and bowel volumes

Patient	1	2	3	4	5	6	7	8	9	10
AJCC stage	II	II	II	II	II	IIIA	IIIA	IIIA	IIIB	IIIB
TNM stage	T2N0	T2N0	T2N0	T3N0	T3N0	T2N1	T4N0	T3N1	T2N2	T3N2
PTV1 volume	2187	2010	1933	2010	2082	1775	1935	1916	2770	2160
PTV2 volume	295	203	141	125	445	293	238	122	279	407
Bowel volume	650	587	606	226	385,5	149	51,5	495,5	450	1011
Bowel-PTV volume	265	219	343	188	268	117	36	302	386	662

For all patients, simulation was performed on computed tomography scan (RT 16 PRO CT Simulator, General Electrics Systems, Cleveland, OH) with a 2.5 mm thick slices from the mid-dorsal spine to the mid-femur. Patients were simulated in the supine position.

The PTV1 included the subclinical and primary disease, with inguinal, perirectal, and pelvic area whereas the PTV2 encompassed the primary disease only. Details of the delineation of these volumes were recently described [5].

The considered organs at risk (OAR) were bowel, bladder, external genitalia/perineal skin (penis and scrotum for men and vulva for women), iliac crests, and femoral heads. For bowel and bladder, a second volume was created and defined as the considered organ minus the PTV (bladder - PTV, bowel - PTV) to avoid hot spots and improve the optimisation. The healthy tissue was defined as the body covered by the CT scan minus the PTV.

The treatment plan was designed to deliver in a single phase process (with simultaneously integrated boost, SIB) a dose of 59.4 Gy to the PTV2 in 33 fractions (1.8-Gy daily fractions) and at the same time 49.5 Gy to the PTV1 (1.5-Gy daily fractions).

Considering the radiobiological equivalent dose, 49.5 Gy in 1.5 Gy fractions were considered to be similar to 45 Gy in 1.8 Gy fractions using the linear-quadratic model and an α/β = 10.

Once the treatment planning was completed, the plan was normalized to cover 99% of the PTV2 with ≥ 95% of the prescribed dose. We also checked that 95% of the PTV1 received 95% of the prescribed dose. No more than 2% of the PTV was allowed to receive more than 107% of the prescribed dose.

### Planning techniques and objectives

Three sets of plans were generated and compared for this study. All IMRT and RA plans were done using 18MV photons using a Varian clinac with a 120 leaves Millennium dynamic multileaf collimator (21 EX, Varian, Palo Alto, CA).

### IMRT plans

IMRT plans were generated using commercial inverse planning software (Eclipse, Helios, version 7.2.34, Varian, Palo Alto, CA). Beam geometry consisted of seven coplanar fields for the whole pelvis with the following gantry angles: 0°, 45°, 110°, 180°, 250°, and 315°. Default smoothing values were used during optimisation. First optimisation criteria and constraints are detailed in our recent publication [5]. Dose rate (DR) of 300 MU/min was selected rather than 600 MU/min, in order to decrease mechanical constraints for multileaves collimator (MLC), even if dosimetric results are similar. Calculation was performed with AAA algorithm, and grid of 2.5 mm.

### RapidArc plans

RapidArc optimisation was performed with the version 8.6.05 from Eclipse, (Helios, Varian, Palo Alto, CA). The maximum DR of 600 MU/min was selected. Starting optimisation constraints consisted in the results of IMRT plans. RapidArc with 1 arc (RA1) corresponded to a single 360° rotation and RapidArc with 2 arcs (RA2) to two coplanars arcs of 360° sharing the same isocenter and optimised independently and simultaneously. These two arcs were delivered with opposite rotation (clock and counter-clock) and so minimize the off-treatment between the two beams time about 25 seconds.

For RA1, field size and collimator rotation were determined by the automatic tool from Eclipse to encompass the PTV. We controlled that the collimator was always rotated to a value different from zero in order to avoid tongue and groove effect.

For RA2, the first arc was similar to the one defined in the RA1 process except for the rotation of the collimator, which was 360-X for the second arc (X corresponded to the rotation of the collimator of the first arc).

To improve the results, we tried to modify constraints and priority factors on IMRT and RA plans. These parameters were modified in function of DVH results for each patient.

When necessary, field size was minimized to 15 cm in the X direction. This dimension corresponded of the maximal displacement of a leave in a MLC Bank. Doing that, all the leaves positions were possible during the optimisation process increasing the degree of modulation even if in beam eye view a part of the volume was excluded of the beam at each gantry position. Globally rotational delivery permitted to irradiate all the volume of the PTV during rotation of the Linac.

### Evaluation tools

Dose Volume Histograms (DVH) were generated to evaluate the three different plans.

For PTV, the parameters D2% and D98% were used as surrogate markers for maximum and minimum doses. Mean dose (D mean) was also reported.

The degree of conformity of the plans was defined as the ratio between the volume receiving at least 95% of the prescribed dose and the volume of the PTV (CI_95%_).

The homogeneity index (HI) was expressed by D5% - D95% (difference between the dose covering 5% and 95% of the PTV).

For all patients DVH for OAR (bowel, bladder, femoral heads, and ilac crests) were calculated and compared. A set of Vx values and D mean was therefore reported.

For healthy tissue, we detailed the volume of the body minus PTV receiving low doses (V5, V10, and V20 Gy).

The number of Monitor Units per fraction required for each plan and the treatment delivery time (from start to the end of the irradiation) was reported.

Treatment techniques were compared using a Mann and Whitney test with significant differences at the p < 0.05 level.

## Results

### PTV volumes, Target coverage, conformity, and dose homogeneity

The mean PTV1 and PTV2 were 2048 ± 305 cc (range, 1619-2770) and 255 ± 112 cc (range, 122-445), respectively. For the bowel, the mean volume was 461 ± 279 cc (range: 51.6-1011.4). Table 1 shows the different volumes (PTV, bowel, and bowel-PTV) delineated for the 10 patients and the results for PTV in terms of coverage and conformity are listed in Table 2.

**Table 2:** Dosimetric results for PTV1 (49.5 Gy) and PTV2 (59.4 Gy)

	PLANS	IMRT	RA1	RA2
PTV1	D98%, Gy (%)	47.1 ± 0.8 (95.2)	46.6 ± 1.2 (94.2)	46.3 ± 0.9 (93.7)
	D95%, Gy (%)	47.7 ± 0.8 (96.4)	47.8 ± 1.2 (96.6)	47.4 ± 0.8 (95.8)
	D2%, Gy (%)	59.8 ± 0.7 (120)	61.6 ± 1.6 (124)^a,b^	60.1 ± 1.3 (121)
	HI, Gy (D5%-D95%)	11.2 ± 0.6	12.8 ± 1.1^a^	15 ± 1.4
	CI	1.2 ± 0.1	1.2 ± 0.1	1.17 ± 0.1
	Dmean, Gy (%)	52.1 ± 1.5 (105)	53.32 ± 1.7(107)	52.2 ± 1 (105)
				
PTV2	D98%, Gy (%)	56.8 ± 0.13 (95.6)	57 ± 0.2 (95.9)^a,b^	56.8 ± 0.1 (95.6)
	D95%, Gy (%)	57.4 ± 0.3 (96.5)	57.8 ± 0.5 (97.4)^a,b^	57.4 ± 0.3 (96.5)
	D2%, Gy (%)	60.6 ± 0.98 (102)	62.7 ± 1.64 (105)^a,b^	60.8 ± 1.3 (102)
	HI, Gy (D5%-D95%)	3.0 ± 0.7	4.3 ± 1.3^a,b^	3.21 ± 1.16
	CI	1.2 ± 0.21	1.8 ± 0.5^a,b^	1.15 ± 0.15
	Dmean, Gy (%)	58.9 ± 0.4 (99.2)	60.3 ± 1.1 (101.6)^a^	59.2 ± 0.9 (99.7)

a if the difference with IMRT is significant

b if the difference with RA2 is significant for RA1

PTV: Planning Target Volume, Dx%: Dose received by x% of the volume, HI: Homogeneity Index, CI: Conformity Index, D mean: Dose mean

All the planning objectives were achieved with the three plans. RA1 reached higher values for the maximum significant dose (D2%) compared to IMRT (p = 0.004) or RA2 (p = 0.01).

**Figure 1:**
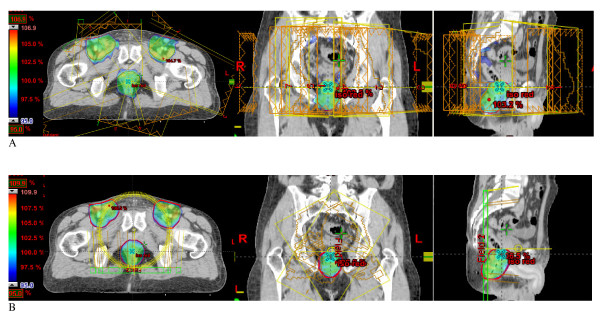
Dose distribution by A: Intensity Modulated Radiation Therapy (IMRT) and B: Volumetric Modulated Arc Therapy (VMAT) RapidArc*.

RA2 and IMRT were equivalent for conformity and homogeneity index with a non significant trend for better results with RA2 (p = 0.09). RA1 showed to be slightly inferior to both IMRT and RA2 for these indices (p < 0.05). Figure 1 depicts dose distribution for IMRT and RA2.

### Organs at risk

Table 3 details numerical findings.

**Table 3:** Dosimetric results for organs at risk

organs	plans	IMRT	RA1	RA2
bowel	Dmean (Gy)	30.4 ± 7.8	29.9 ± 8.4	27.8 ± 7.3
	V30% (cc)	70 ± 26.5 (343 ± 217)	70 ± 27 (343 ± 268)	61.8 ± 24.8 (314 ± 255)
	V40% (cc)	47.7 ± 29.5 (248 ± 182)	49.5 ± 23 (252 ± 203)	42.7 ± 20.8 (224 ± 118)
	V45% (cc)	35.3 ± 21 (193 ± 155)	36.4 ± 19.8 (192 ± 159)	31.5 ± 17.7 (171 ± 150)
	V49.5% (cc)	9.8 ± 15.8 (63 ± 95)	14.9 ± 15.6 (84 ± 99)	10.4 ± 11 (58 ± 82
				
bladder	Dmean (Gy)	39.1 ± 4.2	38.8 ± 4.2	37.5 ± 5.4
	V30%	81.9 ± 15.1	80.4 ± 13.6	70.3 ± 19.4
	V40%	46 ± 17.3	45.6 ± 17.2	40.2 ± 17.2
	V45%	30.8 ± 17	30.1 ± 16.1	26.9 ± 15.5
				
genitalia	Dmean (Gy)	24.5 ± 10.9	19.4 ± 7.3	18.9 ± 6.9
	V20%	62.1 ± 33	51.3 ± 31.6	48 ± 31
	V30%	21.7 ± 22.4	11.8 ± 17.3	9 ± 15.1
		2.3 ± 6,2	1.7 ± 4.9	1.2 ± 3.6
				
Iliac crests	Dmean (Gy)	20.4 ± 7.2	20.6 ± 5.2	19.6 ± 4.4
	V10%	55.3 ± 15.7	60.1 ± 15.9	58.9 ± 18.2
	V20%	37.6 ± 9,2	45.25 ± 13.9	40.6 ± 10.5
				
Femoral heads	D mean (Gy)	27.5 ± 2.4	26.2 ± 2.2	24.6 ± 4.1
	V45%	2.2 ± 3,3	2.2 ± 2.3	1.7 ± 1.9
	Dmean (Gy)	27.2 ± 2.4	26.85 ± 2.9	24.1 ± 3.2 ^a^
	V45%	0.8 ± 1	1.7 ± 1.9	1.3 ± 1.5
				
Healthy tissue	V5%	55.7 ± 5.4	57 ± 5.1	57.6 ± 5.1
	V10%	47 ± 5	46.7 ± 6	47.2 ± 5.7
	V20%	31.9 ± 3.9	33.4 ± 7	28.8 ± 4.8

IMRT: Intensity Modulated Radiation Therapy, Vx%: Volume receiving x% of the prescribed dose, D mean: Dose mean a the difference between RA2 and IMRT is significant

The bowel DVH parameters (V49.5 V45, V40, V30) were similar for the three plans. The mean dose was reduced by 4.7 Gy with RA2 compared to IMRT, (IMRT: 38.8 ± 12.9 vs 34.1 ± 17.7 Gy, p = NS).

The plans for the bladder were also equivalent. The volume of bladder irradiated at low and medium doses was reduced with RA2 compared to IMRT with a trend towards statistical significance (p = 0.07).

The same trends were observed for skin and genitalia with a decrease of the irradiated volume to the low and medium doses (V30: IMRT: 21.7 ± 22.4 Gy vs RA2: 9 ± 15.1 Gy, p = 0.08). The mean dose reduced by 5.6 Gy (IMRT: 24.5 ± 10.9 vs 18.9 ± 6.9 Gy, p = NS)

Considering the (i) femoral heads and (ii) the iliac crest,(i) the mean dose was reduced by 3 Gy with a significant statistical difference (p = 0.03). V45 was inferior to 5% for all techniques. (ii) No significant difference between the plans was noted for the parameters V10, V20, and mean dose.

Finally, the planning objectives for healthy tissue consisted in minimize the dose, in particular the low doses about 5, 10, and 20 Gy. IMRT and RA plans showed similar results.

**Figure 2:**
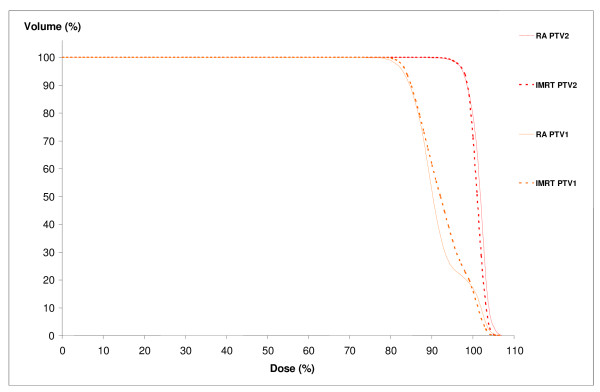
**Dose-volume histograms for PTV**. RA, Rapidarc; IMRT, intensity-modulated radiotherapy

**Figure 3:**
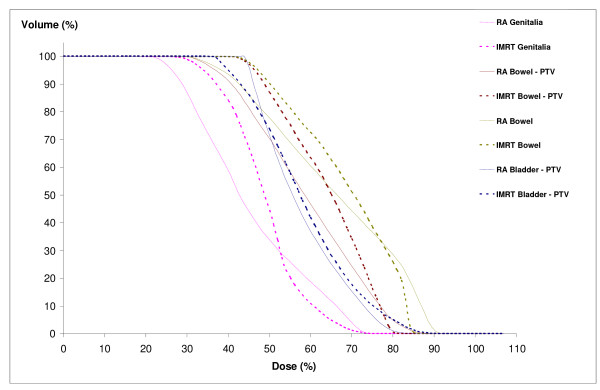
**Dose-volume histograms for bowel, genitalia, and bladder**. RA, Rapidarc; IMRT, intensity-modulated radiotherapy

**Figure 4:**
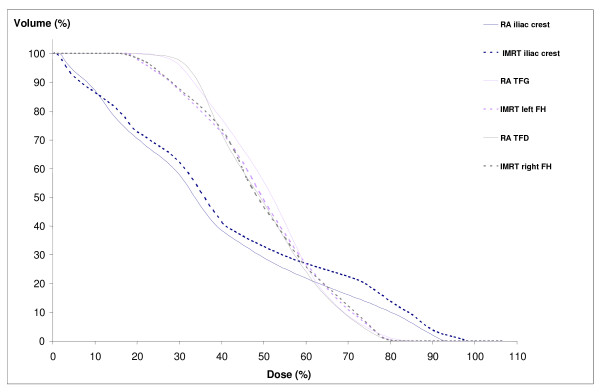
**Dose-volume histograms for iliac crests and femoral heads. **RA, Rapidarc; IMRT, intensity-modulated radiotherapy; FH, femoral head

We reported DVH results for one patient in figures 2, 3 and 4.

### Monitor Units and Delivery time

The IMRT plans required a mean of 1646 ± 332 MUs per fraction whereas the RA1 plans required 80% less (330 ± 52 MUs, p = 0.0002). The use of two arcs resulted to an slight increase of the number of MU (493 ± 66) compared to one arc (p = 0.0003). The difference between RA2 and IMRT remained significant (p = 0.0013).

Compared to a delivery in 14 min for IMRT, treatment time (defined as the start to the end of the irradiation) with RA was definitely shorter and was 1.1 and 2.3 minutes for one and two arcs, respectively.

## Discussion

Recent progresses of new technologies in RT are of great interest for quality of treatment and avoidance of toxicities for miscellaneous localizations, in particular for anal canal cancer.

We initially performed a dosimetric study to compare standard RT3D with IMRT showing a significant decrease of the irradiated volume of the OAR, especially for the bone marrow, while keeping an excellent coverage of the PTV [5]. Based on these results we decided to treat patients suffering from anal canal cancer with IMRT as a standard with promising results in terms of acute toxicities [7-10]. RapidArc is a promising technique, providing a coverage of the target volume and spare of organs at risk at least equivalent to IMRT, while it could reduce significantly the treatment time and the number of MU required [14-22].

In the present study, RapidArc proved to be equivalent to IMRT for targeting coverage of anal canal but showed better organ sparing in terms of mean dose. We also confirmed that RapidArc with two arcs (RA2) achieve better results than 1 arc (RA1) for consequent or complex target volume in terms of conformity and homogeneity [14,17]. Two arcs allowed superior modulation factor during optimisation due to the independent optimisation, and unrelated sequence of MLC shape, gantry speed and dose rate combinations. Some particular options have to be considered for better modulation compared to one arc, particularly the decrease of the MLC in the X direction allowing the optimization process. This approach allows better homogeneity un the target volume and erases the hot spots outside the PTV compared to IMRT (Figure 1).

Clivio et al.[14] published also similar results for PTV but showed different benefit for the OAR, namely for the bowel tract. In one hand the PTV volumes are higher in our series (PTV1 minus PTV2 = 1793 ± 283 cc vs 1307 ± 355 cc) due to a 1-cm margin from the CTV to the PTV (8 mm for Clivio et al.). We also delineated the lymph node CTV as recommended by Taylor and al. [23] using a margin of 1 cm around the vessels. In the other hand, we found lower bowel volumes (461 ± 279 cc vs. 2483 ± 774 cc). Indeed, Gallagher et al. estimated that 1/3 of the small intestine volume would correspond to 660 cc leading to a maximal entire volume of 2000 cc [24].

Our mean irradiated volume above 30 Gy was about 314 cc with RA2 but 844 cc in the Clivio et al. report [14]. Regarding correlation between dosimetric parameters and acute toxicity, Devisetty et al. [9] showed higher acute GI toxicity for V30 > 450 cc and ≤ 450 cc (33% vs. 8%, p = 0.003, respectively).

Regarding acute hematologic toxicity, Mell et al. suggested an association between dosimetric parameters for iliac crests and acute hematologic toxicities, especially in the range of low doses (10 and 20 Gy) [25-27]. We did not find a significant difference between IMRT and RA for these values shown acceptable when compared with 3D [5]. Other dosimetric parameters were also interesting with constraints for bone marrow given for higher doses in the RTOG 0529 protocol (V30, V40, and V50) [28] showing only 23% grade 3-4 leucopenia [10].

Considering late toxicities, we referred to the publication of Emami et al. [29] asking for a TD 5/5 (probability of developing 5% of chronic toxicity within 5 years) for bowel, bladder, and femoral heads. Long-term follow-up are warranted before drawing definitive conclusions.

One of particular interest of RapidArc is the reduction of the time to deliver each fraction and the number of required MU [14,15,18,30]. IMRT plans presented in this study were wider than 15 cm in the direction of the MLC motion necessitating splitting into 2 sequences and doubling the number of fields. Dose rate about 600 MU/min for IMRT would reduce the beam on time, but not the effective treatment time, which is mainly due to the multiples beams and the "off-time" necessary to move from one to another. The number of MU required is higher due to the sliding window technique. A "step and shoot" technique might lead to lower values. By contrast, treatment with RA is performed simultaneously with rotation by a dynamic MLC adaptation to the target structure during the rotation (the open surface is more important than for sliding window) which reduces the number of MU. For two arcs, the rotation in clock and counter-clock directions allows minimized off-time (25 seconds between the 2 arcs). There is no doubt that the reduced treatment time may impact on the treatment quality avoiding long and uncomfortable treatment for the patient and reducing the risk of internal organ motion during the fraction. In addition, more time can be spared for on-line control imaging. A such prolonged fraction delivery time may also have an impact on treatment outcome, due to the increase in cell survival by recovery from sub lethal damage [31,32].

One of the downfalls of IMRT is the potential risk of second cancer [33-36]. Theoretically, the significant reduction of MU with RapidArc decreases scattered dose and may reduce the risk of secondary malignancy. The impact of irradiation of healthy tissue at low doses remains unresolved with the use of RapidArc even if this technique is capable to reduce medium and integral body doses [15,30].

## Conclusion

Compared to IMRT, RapidArc provides an equivalent coverage of PTV and OAR sparing while reducing the number of MU and the treatment time delivery. These improvements have led us to implement rapidly this technique into the clinic. Nine patients have already been treated with RapidArc using two arcs.

## List of abbreviations

3D-CRT: three-dimensional conformal radiation therapy; CI: Conformity Index; CTV: Clinical Target Volume; D mean: Dose mean; DVH: Dose-volume histograms; DR: Dose rate; HI: Homogeneity Index; IMRT: intensity-modulated radiation therapy; MLC: Multileaf collimator; MU: Monitor units; OAR: Organs at risk; PTV: planning target volume; RA: RapidArc; SIB: simultaneous integrated boost; VMAT: Volumetric Modulated Arc Therapy, Vx%: Volume receiving x% of the prescribed dose

## Competing interests

The authors declare that they have no competing interests.

## Authors' contributions

SV, PF conceived the study, collected data, and drafted the manuscript. NA, CLM, JBD, CL, and DA participated in coordination and helped to draft the manuscript. SG performed the statistical analyses. DA provided mentorship and edited the manuscript. All authors have read and approved the final manuscript.
